# A video game based hand grip system for measuring muscle force in children

**DOI:** 10.1186/s12984-021-00908-1

**Published:** 2021-07-10

**Authors:** Orla Gotthelf, DeWayne Townsend, William Durfee

**Affiliations:** 1 Department of Mechanical Engineering, University of Minnesota, Minneapolis, USA; 2 Department of Integrative Biology and Physiology, University of Minnesota, Minneapolis, USA

**Keywords:** Muscular dystrophy, Muscle force assessment, Game-playing, Muscle fatigue

## Abstract

**Background:**

While new therapies are continuously introduced to treat muscular dystrophy, current assessment tests are challenging to quantify, cannot be used in non-ambulatory patients, or can de-motivate pediatric patients. We developed a simple, engaging, upper-limb assessment tool that measures muscle strength and fatigue in children, including children with muscular dystrophy. The device is a bio-feedback grip sensor that motivates children to complete maximal and fatiguing grip protocols through a game-based interface.

**Methods:**

To determine if the new system provided the same maximum grip force as what is reported in the literature, data was collected from 311 participants without muscle disease (186 M, 125 F), ages 6 to 30, each of whom played the four minute grip game once. We compared maximum voluntary contraction at the start of the test to normative values reported in the literature using Welch’s unequal variances *t*-tests. In addition, we collected data on a small number of participants with muscle disease to determine if the assessment system could be used by the target patient population.

**Results:**

Of the 311 participants without muscle disease that started the test, all but one completed the game. The maximum voluntary contraction data, when categorized by age, matched literature values for hand grip force within an acceptable range. Grip forced increased with age and differed by gender, and most participants exhibited fatigue during the game, including a degradation in tracking ability as the game progressed. Of the 13 participants with muscle disease, all but one completed the game.

**Conclusions:**

The study demonstrated the technical feasibility and validity of the new hand grip device, and indicated that the device can be used to assess muscle force and fatigue in longitudinal studies of children with muscular dystrophy.

## Background

Muscular dystrophy is a group of genetic muscle diseases marked by progressive loss of muscle mass and muscle weakness [1]. Duchenne muscular dystrophy (DMD) is the most common form and is a genetic defect characterized by an absence of dystrophin, a protein that maintains the structure of muscle fibers [2]. DMD is an X-linked genetic disorder that primarily affects boys and birth prevalence of DMD among males is estimated at 1 in 5000 [3].

DMD is diagnosed early and progresses steadily [4]. Onset is between two and seven years and a wheelchair is typically needed sometime between the ages of 10 and 14. By the late teens, there is significant loss of upper extremity strength, including the ability to move the arms. Today, with advances in respiratory and cardiac care, many with DMD live into their thirties, although most do not survive past the age of 25 with death typically resulting from respiratory complications. While there is no cure for muscular dystrophy, therapies can manage symptoms and slow the evolution of the disease [5–8]. For example, corticosteroids have been shown to moderate disease progression, but are not universally prescribed because of side effects [7, 9].

For clinical care of a patient with DMD or for any clinical trial examining new DMD treatments there is a need for quantitative assessment measures to precisely track the progress of the disease or to measure a treatment effect. A sensitive and reliable quantitative assessment tool is needed by clinicians who must track and record the status of their DMD patient to assess a response to therapy. It is needed by clinicians and parents who are seeking full knowledge of a child’s clinical status and how that status has changed over time. In addition it is most acutely needed for clinical trials that require a highly reproducible and quantitative assessment of muscle function to determine the therapeutic effect of new treatments.

With the recent and robust history in developing candidate drugs to treat DMD, there has been considerable attention paid to what outcome measures should be used in clinical trials. Examples, some of which are described below, are reported in [10–23] as well as in recent reviews [23–26]. All of these measures have flaws that limit their usefulness for clinical assessments. The device we describe in this paper addresses many of these flaws and is anticipated to provide a reliable quantitative assessment of muscle function in the pediatric DMD patient population.

Because DMD is a muscle disease, the majority of outcome measures test skeletal muscle function. The most common test used is the manual muscle test (MMT), which is performed informally in practically every clinic visit by a DMD patient and performed formally in many DMD clinical studies. For the MMT, the patient voluntarily contracts the muscle against a gravity load or resistance with the examiner determining the response on a 0–5 scale [27, 28]. While easy to learn and easy to apply, the reliability, accuracy, and inter-rater reliability of MMT has been questioned [27, 29, 30]. Further, MMT cannot be used to detect small changes in strength seen in a relatively slowly progressing disease such as DMD [20, 31].

Quantitative muscle testing (QMT) measures maximum voluntary contraction using a mechanical or electronic dynamometer that records the reaction force. The quantitative output improves test-retest and inter-rater reliability compared to MMT so long as a standardized protocol is used [20, 32–34]. Systems such as The Richmond Quantitative Measurement System and Kin Com’s 125 are examples of more complex QMT devices that have been used in DMD and spinal atrophy studies to measure the strength of several muscles [19, 20, 22, 35]. These systems, however, are costly and require a bulky fixation apparatus which limits their utility in the clinic and in clinical trials.

A common outcome measure used in many DMD studies is the six-minute walk test (6MWT) where the patient walks as far as possible in six minutes on a walking course 30 m in length [36, 37]. A modified version for children has been developed for use in DMD patients and has been shown to be safe, easy to apply and reproducible [11–13]. The primary limitation of the 6MWT is that it requires ambulation, which means it cannot be used for long term studies or for assessing patients in the later stages of DMD who are in a wheelchair. Further, the patient’s motivation to perform the test can impact performance.

The North Star Ambulatory Assessment (NSAA) is a 17 item test of functional abilities that are important for ambulation [14]. The NSAA has been shown to be reliable and to correlate with other measures such as the 6MWT [15–18]. The NSAA can be difficult to quantitate and is limited to patients who are ambulatory. Also the complexity of the NSAA tasks can lower motivation to perform, especially in younger patients, which in turn can influence the score.

For non-ambulatory DMD patients, a recent study concluded that a suite of functional measurements could be used for outcome assessment but also pointed out that there is no consensus on which measures are best [21]. A review of assessment tools for upper limbs also highlighted the need for measures that can be used over the full spectrum of DMD as studies only using 6MWT or NSAA measures cannot follow children who have lost ambulation during the course of the trial [25].

The Performance of the Upper Limb (PUL) is a recent assessment tool that was created specifically for measuring upper limb function in ambulant and non-ambulant patients with DMD [38], and was shown to be reliable in a multi-center trial and to be a useful measure when combined with the 6MWT for ambulatory boys [39, 40].

Hand grip is commonly measured in DMD studies and is a good indicator of muscle strength [21, 41]. Generally, grip is measured with a handheld tool such as the Jamar dynamometer, for which there are normative values for children [42] and adults [43]. While hand weakness can be detected in the early stages of DMD, it is only revealed by comparing to control values as the weakness from the disease is counteracted by the normal hand strength that increases in the first decade [41]. Because grip strength can be measured for ambulant and non-ambulant DMD patients and because it is fast and easy to apply, it is useful for short and long-term assessment of muscle function in a child with DMD. Traditional hand grip testing depends on patient motivation, which in turn relies on patient engagement. This can be a challenge with a test that uses a hand dynamometer as children with progressive disease can see that their grip strength is decreasing with age.

The standard hand grip test only measures maximum voluntary contraction as an indicator of muscle function. For assessing outcomes related to DMD we believe there may be value in also measuring muscle fatigue as fatigue is prevalent in DMD patients along with loss of strength [4]. In animal models of DMD and in DMD patients there is evidence of defects in the regulation of blood flow in response to contraction [44, 45], which results in the development of functional ischemia, the consequences of which are demonstrated in the extreme levels of fatigue evident in mouse models of DMD following strenuous exercise [46]. Fatigue is commonly reported in patients with DMD [47], but the standard tests reviewed here, including MMT, QMT and the 6MWT, are unable to reliably measure fatigue in DMD patients [48].

Thus there is a need for a simple, engaging upper-limb assessment tool that measures muscle strength and fatigue in children with DMD. In this paper we describe such a device: a game-based hand grip measuring system that is quantifiable, can be performed by patients at all levels of muscle disease progression, including non-ambulatory patients, and is fun and motivating for the patient. We describe how the device works and report on the results of a study where the grip strength of people who do not have muscle disease was measured using our device. Our study demonstrates that the new device reports hand grip strength that is consistent with grip strength reported in the literature from normative studies using a traditional grip dynamometer. In addition we report on the use of the measuring system by a small number of patients with neuromuscular disorders, almost all of whom were able to complete the test.

## Methods

### Hand grip system

The hand grip force measuring system consists of two components: a hand-grip dynamometer that fits into the hand and contains force sensors to measure grip force along with associated sensor conditioning electronics, and a computer-based bio-feedback game that the user plays by squeezing the dynamometer.

**Fig. 1:**
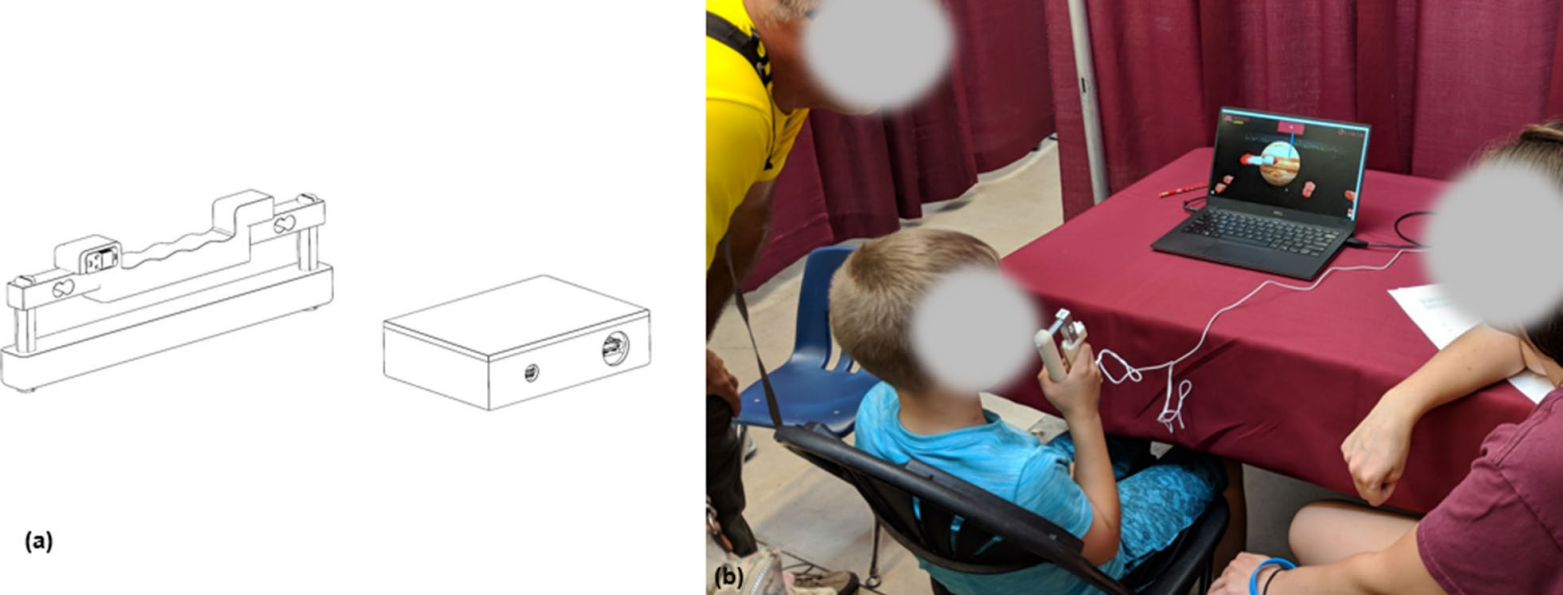
**a** Hand-grip dynamometer and associated electronics interface box. **b** System in use at the 2018 Minnesota State Fair

The dynamometer (Fig. 1) has two plastic bars bridged by a pair of load cells. The low-force model uses 20 kg load cells (Phidgets 3134_0) and can measure up to 40 kg while the high-force model uses 50 kg load cells (Phidgets 3135_0) and can measure up to 100 kg. Squeezing the bars together causes grip force to be transferred to the sensor whose output is filtered, amplified, and digitized (24-bit at 80 Hz, SparkFun SEN-13261), and then sent to a laptop via a USB connection. The design was intended to be as light weight as possible so that it could be used by children of all ability.

The dynamometers were calibrated using a load frame (MTS QTest QT/10). The devices were compression loaded at a rate of 0.2 mm/s to a load of 300 N for the low force model and 700 N for the high force model. Regression lines were fit to the force-time data from the load frame and the force-time data from the grip sensor and the line slopes divided to generate a calibration constant. The load test was repeated three times to produce an average calibration constant for the device.

**Fig. 2:**
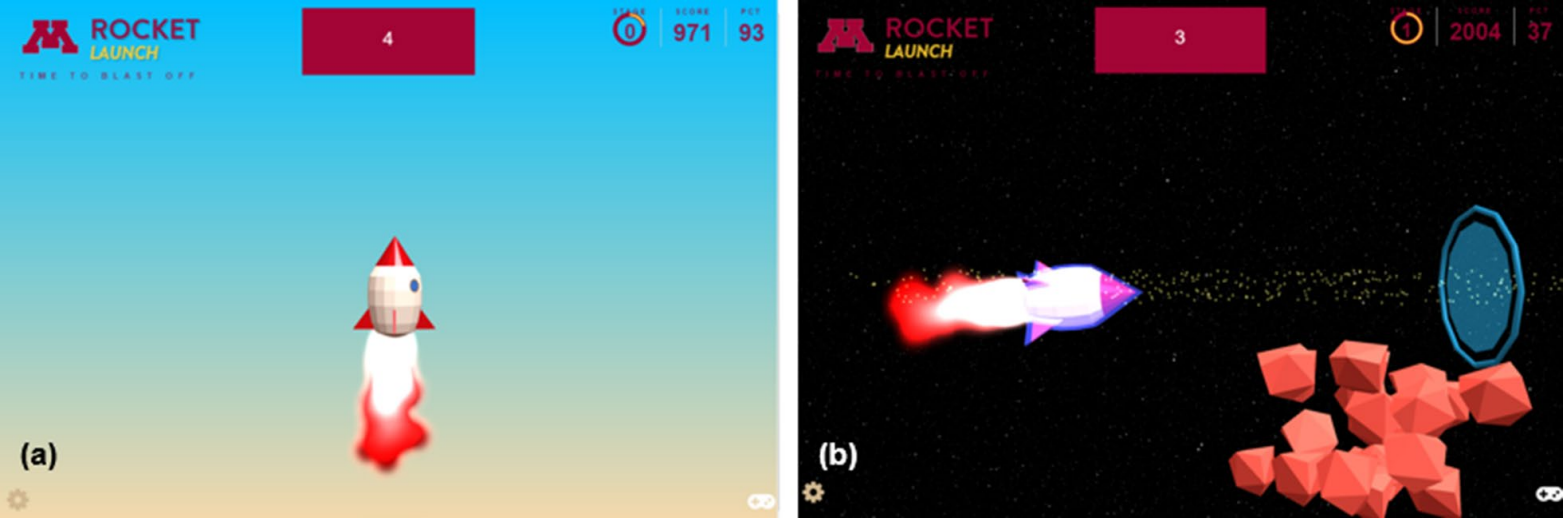
**a** Screen shot of the calibration task from the Rocket Launch application. **b** Screen shot of the tracking task from the Rocket Launch application

Grip strength was tested by having participants play the Rocket Launch video game, which uses the hand-grip dynamometer as a controller. Squeezing and relaxing the dynamometer causes an animated rocket to move up and down on the game screen (Fig. 2). As participants play the game, they are taken through an exercise protocol. For participants, the goal of the game is to score points by staying above the asteroid belt and by flying through the rings that periodically appear (Fig. 2B). The user watches the screen to determine the desired path to earn points. The higher on the screen that the asteroid belt and rings appear the harder the participant must squeeze the dynamometer to earn points. The Rocket Launch application allows the researcher to construct the game exercise protocol by specifying any combination of calibration periods, tracking periods where the user squeezes the handle to move the rocket to the desired path, and rest periods, each of any length. During a calibration period, the user is instructed to squeeze the handle as hard as they can, which causes to rocket ship to launch into the air (Fig. 2A). The initial pre-game calibration period establishes the maximal voluntary contraction (MVC) for grip force, which is used to scale the target forces during the asteroid belt tracking periods. The game is connected to the cloud through a secure link. Game data is stored in a secure database and game parameters can be adjusted remotely, which means that any local laptop can be used to run the game.

**Fig. 3:**
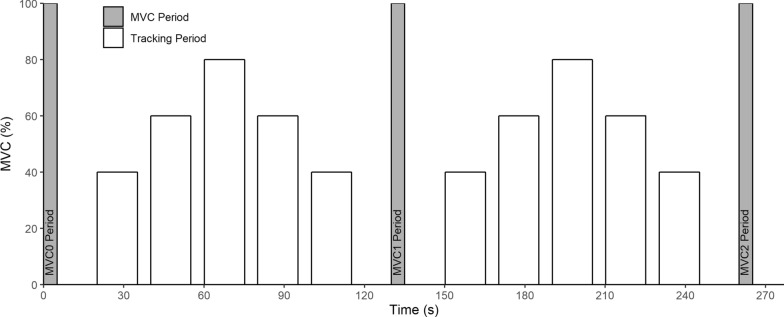
Task interval time line used in the Rocket Launch game

The game used to collect data for this study was 265 s in duration with 5 s calibration periods at the beginning, middle and end of the game. Following the initial calibration to gather MVC, there was a 15 s rest period then tracking periods of 40%, 60%, 80%, 60%, and 40% of the initial MVC, each lasting 15 s and followed by 5 s rest periods between each period. Following the last 40% tracking period was a 15 s rest period. Next came the mid-game determination of MVC. A repeat of the tracking and rest periods followed and the game terminated with a determination of the post-game MVC. Figure 3 shows the timeline for this game.

### Validation study

To validate the grip device as a muscle force assessment tool, we ran a study using participants without muscle disease. The study was run at the University of Minnesota Driven to Discover Research Facility at the 2018 Minnesota State Fair, which is among the largest state fairs in the United States. The study was approved by the University of Minnesota Institutional Review Board (IRB ID: STUDY00003448).

#### Participants

Participants were recruited from visitors to the fair. The inclusion criterion was to be in the age range of 6 to 30 years. Exclusion criteria were having an identified neuromuscular disease diagnosis or being unable to follow directions.

#### Apparatus

The hand grip dynamometer and Rocket Launch video game described above were used to assess muscle force.

#### Procedure

All activities took place during a single 10-min session. Participants or their parents self-reported their age and gender. The participant was seated in front of the screen and instructed to hold the dynamometer with their dominant hand, their elbow bent approximately 90 deg and their wrist in the neutral position. Younger children used the low-force model dynamometer while the rest used the high-force model. Following a brief instruction period, the participant played the Rocket Launch game, with the game protocol described above. During the three calibration periods, the researcher urged the participant to squeeze as hard as possible (to “launch the rocket”). During the tracking periods positive feedback was provided when the participant was able to earn points by tracking the target.

### Data analysis

Maximum voluntary contraction during the beginning (MVC0), middle (MVC1), and end (MVC2) of the game was determined by finding the maximum grip force during the calibration period and calculating the average of a five point range starting two data points prior to the maximum value. If the maximum force was greater than 125% of that average the data set was flagged as containing an outlier and was not used for further analysis. (Outliers were present in less than 1% of the data sets).

During grip force target tracking, root mean square (RMS) tracking error (the difference between the desired trajectory indicated by the location of the rings and the actual trajectory of the rocket) was calculated for each sample using

1 $$RMS = \sqrt{(Target \% - Response \%)^2}.$$

The total RMS tracking error was the sum of the sample-by-sample error over the period of interest.

Summary statistics (mean, standard deviation (SD), and range) were calculated for age group and gender. The Pearson correlation coefficient was used to measure the correlation between MVC and age and tracking error, and a linear regression model to determine the $R^2$ value between MVC and age. ANOVA with post hoc Bonferroni correction was used to determine if there were any differences between the pre-game, mid-game and post-game MVC readings. A paired *t*-test was used to determine if there was any difference between tracking error during the 80% MVC period in the first half of the game and the the 80% MVC period in the second half of the game.

To validate whether the new device produced MVC readings that were the same as those reported in the literature, we compared our data to data from eight previous studies that assessed hand grip strength in pediatric populations [42, 43, 49–54]. These studies all used a commercially available hand grip dynamometer with six of the eight using the gold-standard Jamar dynamometer. For the comparisons, participants were grouped by age according to the age groupings used by Mathiowetz [42, 43]. Grip force averages and standard deviations were calculated from the eight studies for each age group and sex. Differences for each age group and sex between the initial MVC (MVC0) reported by our device and the literature-based normative data were determined using Welch’s unequal variances *t*-tests. (The Shapiro-Wilk normality test was run for each age group in our data and the test statistic showed that the MVC0 data points in all groups were approximately normally distributed). All statistical tests were performed using R, version 3.6.3 [55].

### Use by people with muscle disease

To determine if the device could be used by the intended patient population, in addition to the main study we recruited a small number of individuals with muscle disease. This sub-study was approved by the University of Minnesota Institutional Review Board (IRB ID: STUDY00001330). The purpose of this part of the project was to determine whether patients, and particularly children with muscular dystrophy, could understand and complete the grip strength game. The number of participants in the sub-study was designed to determine if participants could complete the game and was not powered to provide conclusions about muscle strength or game performance.

## Results

### Participants

Over four days 311 participants were enrolled in the study. Of those, 29 had missing or incorrect data, one did not finish the game, 39 saturated the dynamometer, three had outliers in the initial MVC data and two had data errors in the mid or post-game MVC readings. This left a sample size of 237 participants for the data analysis.

**Table 1:** Characteristics of participants (Age, Gender), and mean, standard deviation and range of grip strength in dominant hand (*N* = 237)

Age	Males				Females			
	n	Mean (N)	SD (N)	Range	n	Mean (N)	SD (N)	Range (N)
6–7	10	128	47	73–227	7	103	27	69–141
8–9	17	182	50	78–273	9	161	32	98–200
10–11	26	199	56	121–325	12	204	42	142–290
12–13	28	256	72	140–467	17	230	48	155–328
14–15	11	383	138	209–660	12	296	34	232–348
16–17	5	366	119	223–554	10	293	49	216–366
18–19	9	507	155	237–755	4	302	17	291–327
20–24	18	476	80	333–581	15	311	67	169–455
25–29	11	526	139	344–735	10	303	68	165–414
30–34	4	614	129	463–764	2	334	27	314–353
*Total*	139				98			

Of the 237 participants 139 (59%) self-identified as male and 98 (41%) self-identified as female. Table 1 reports the demographics of the participants, mean strength, standard deviation of mean strength, and range of strength, following the age-groups of Mathiowetz [42, 43].

**Fig. 4:**
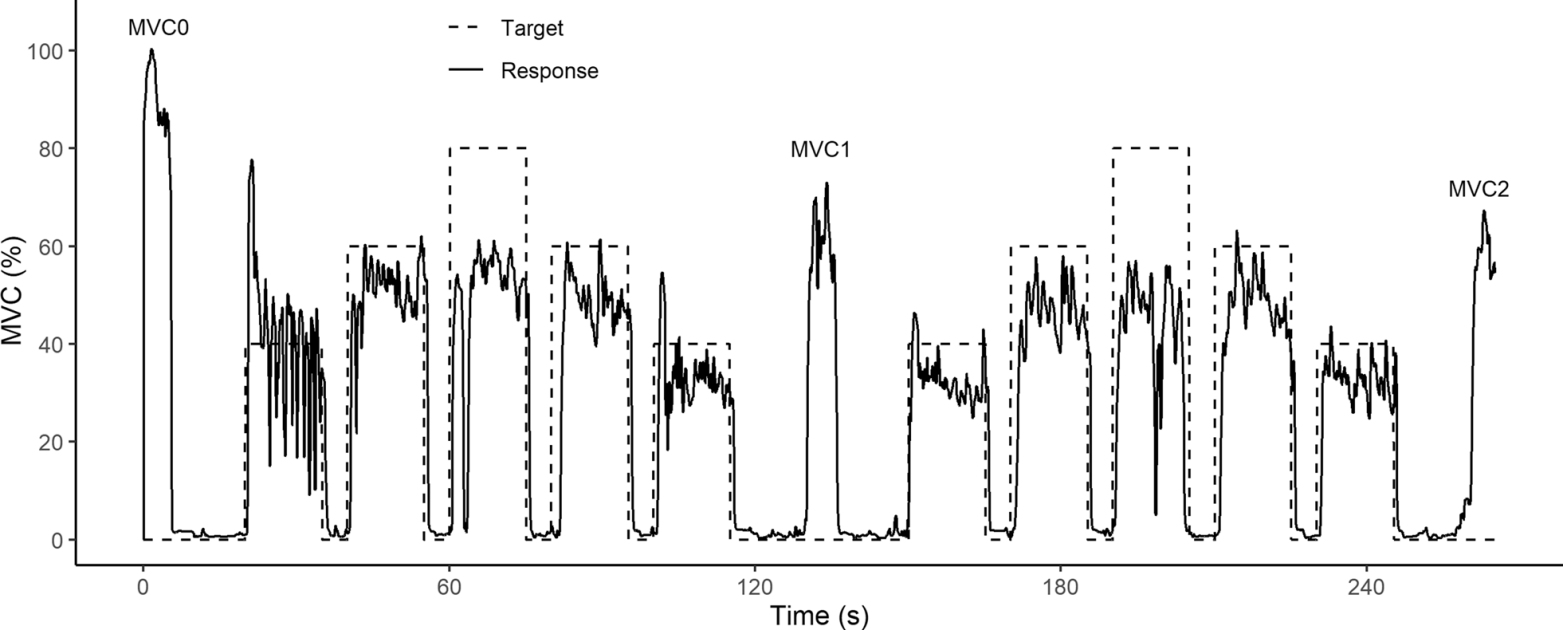
Example of run data from a 9 year old male participant. Target is percent MVC0. MVC0, MVC1 and MVC2 indicate the three MVC tests during the game

Figure 4 shows one example of dynamometer grip force data from one session. The game has three five-s calibration periods: at the start, middle and end of the game, and 10–15-s tracking periods at 40%, 60% and 80% of the initial MVC. The MVC for this participant was lower at the middle and end of the game. The participant had reasonable tracking at the 40% and 60% MVC levels but had difficulty tracking at the 80% level. This participant, like others, had an overshoot during the initial 40% tracking period as they were learning to control the rocket.

### Comparison of MVC to normative data

**Fig. 5:**
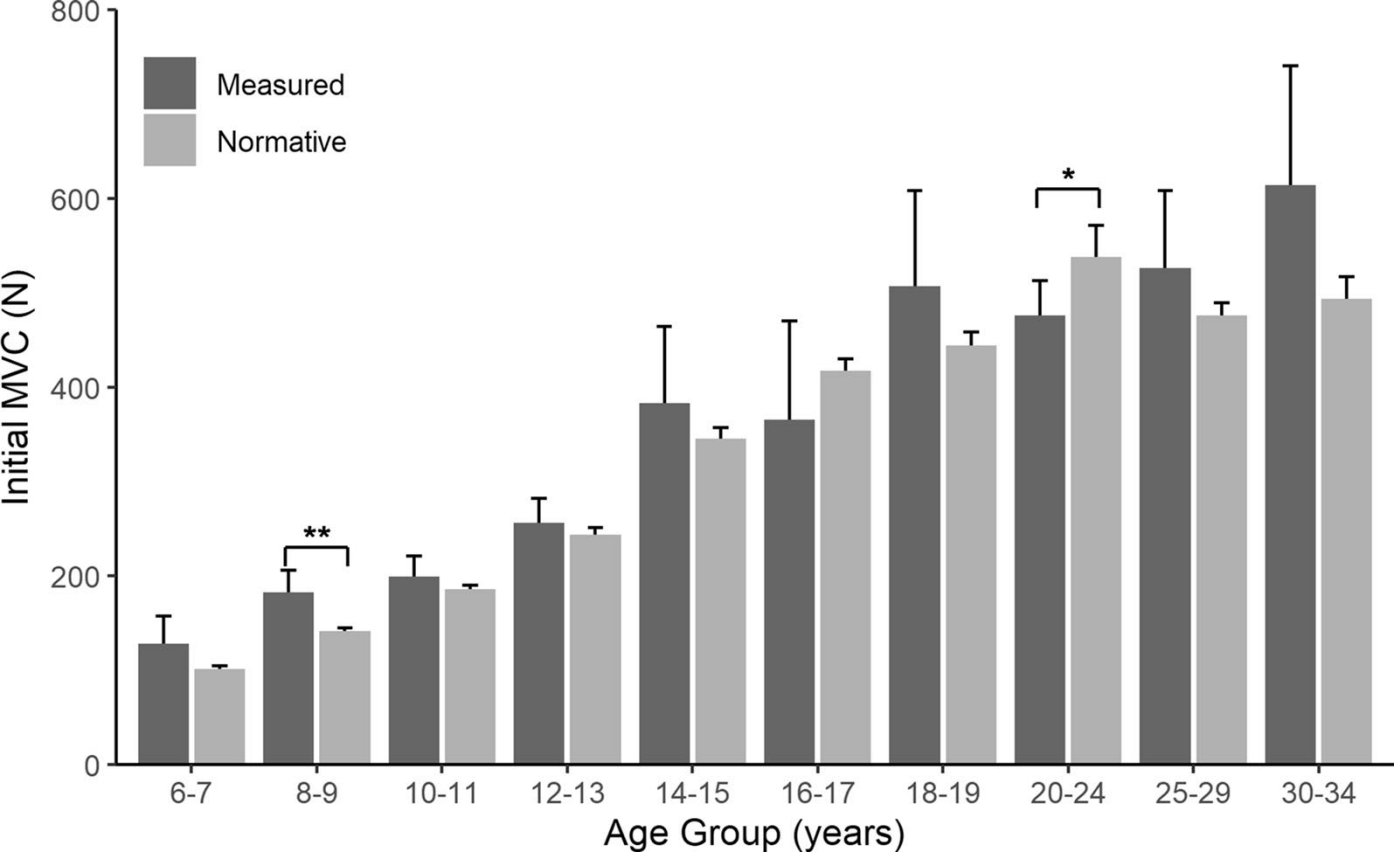
MVC0 (mean+95%CI) of male participants ($\textit{n} = 139$, dark bars) compared to normative male data (light bars) from multiple sources [42, 43, 49–54], grouped by 2 year age ranges from 6 to 19 and 5 year age ranges from 20-34. Significant differences marked by *($\text {p} <.05$) and **($\text {p} <.01$)

**Fig. 6:**
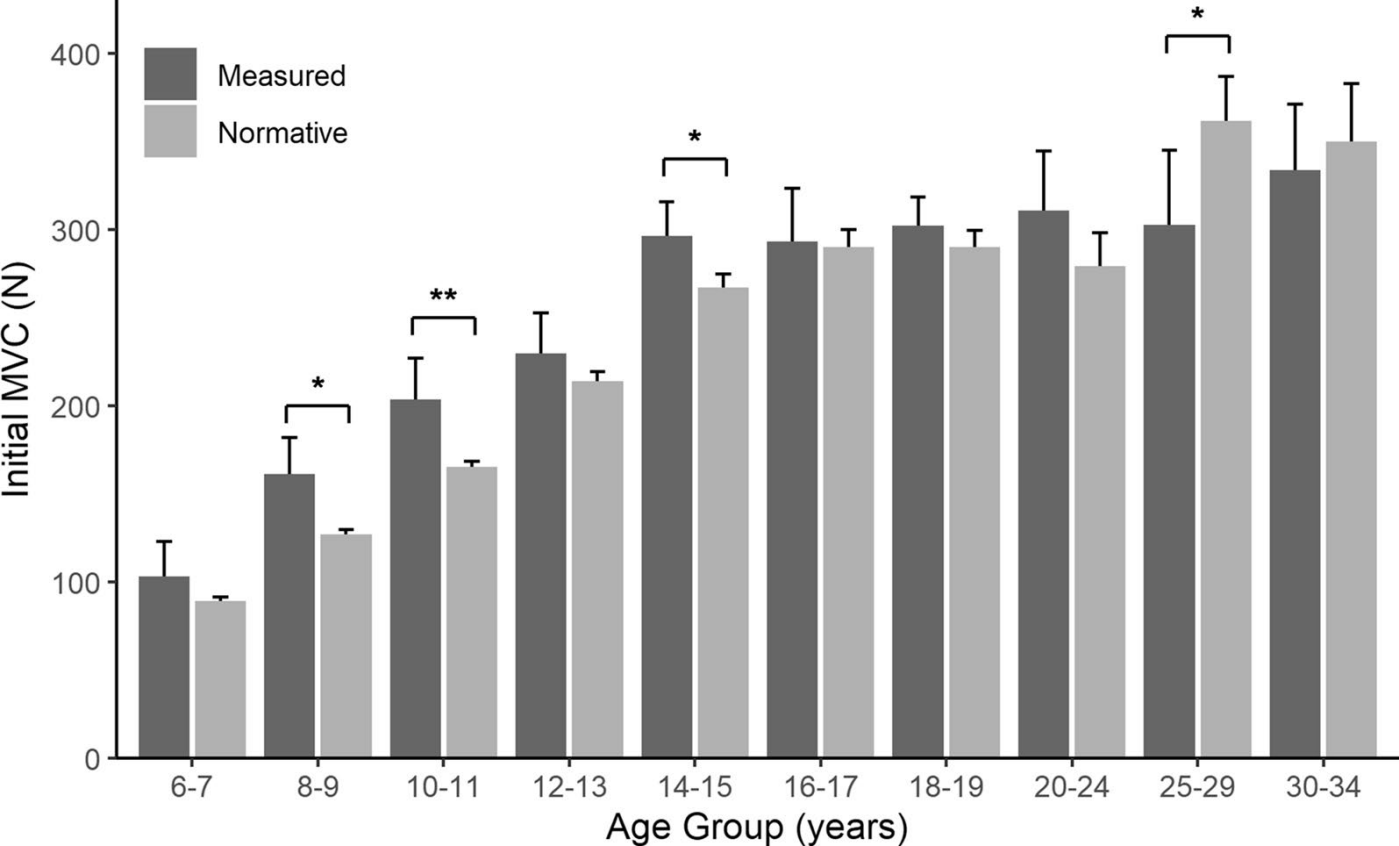
MVC0 (mean+95%CI) of female participants ($\textit{n} = 98$, dark bars) and normative female data (light bars) from multiple sources [42, 43, 49–54], grouped by 2 year age ranges from 6-19 and 5 year age ranges from 20 to 34. Significant differences marked by *(*p* <. 05) and ** ($\text {p} <.01$)

Figures  5 and 6 show how the measured data from this study compares to normative data reported in the literature. For males (Fig. 5) ages 8–9 reported higher average MVC than the normative data ($\text {p} <0.01$) and ages 20-24 reported lower average MVC than the normative data ($\text {p} <0.05$). For females (Fig. 6) ages 8–9, 10–11 and 14–15 reported higher average MVC than the normative data ($\text {p} <0.05$, $\text {p} <0.01$ and $\text {p} <0.05$ respectively) while ages 25–29 reported lower average MVC than the normative data set ($\text {p} <0.05$). The remaining 14 of the 20 age groups had the same average MVC between collected data and the normative data sets.

### Properties of MVC

**Fig. 7:**
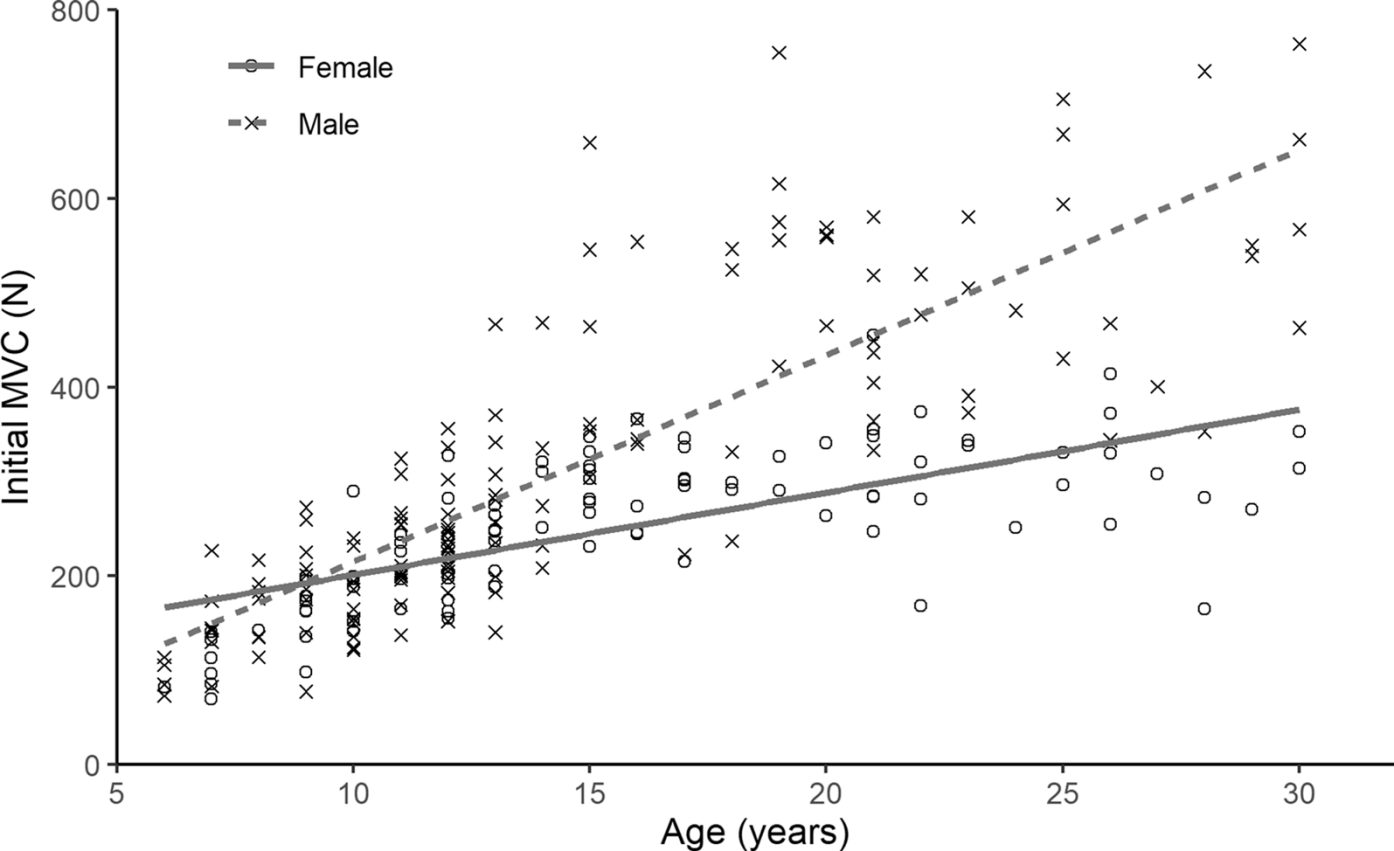
Grip strength by age in a healthy population of males ($\textit{n}=139$) and females ($\textit{n}=98$) with trendlines for male ($r^2=0.67$) and female ($r^2=0.46$). The trendlines show that MVC increases with age for both genders

Figure 7 shows how MVC grip strength varied with the age of the participant. There was an increase in MVC0 as age increased for both male and female participants, which concurs with what is reported in the literature [42, 43, 49–54].

**Fig. 8:**
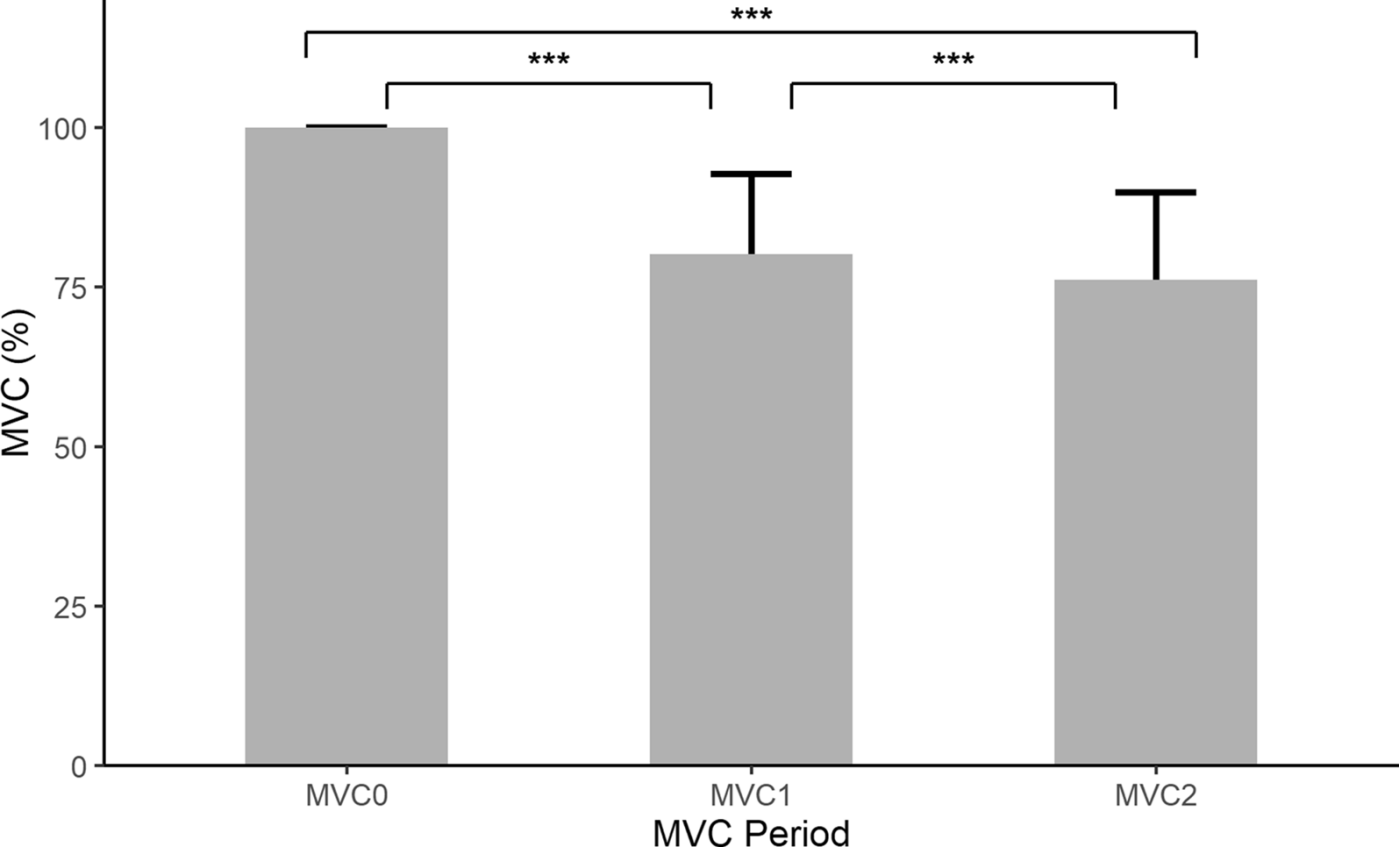
MVC % (mean+SD) during MVC0 ($\text {M}=100.0$, $\text {SD}=0.0$), MVC1 ($\text {M}=80.2$, $\text {SD}=12.6$) and MVC2 ($\text {M}=76.2$, $\text {SD}=13.7$) periods of gameplay in healthy population ($\text {N}=237$ for each group). There was a significant difference between groups, $\text {F}(2, 708)=335.8$, $\text {p} <.001$ with post hoc comparisons using Bonferroni correction indicated that all three groups were significantly different from each other (***$\text {p} <0.001$)

Most participants exhibited muscle fatigue as the game progressed. Figure 8 shows the average MVC across all participants for the pre-game, mid-game and post-game calibration periods. There were differences between the three ($\text {F}(2, 708)=335.8$, $\text {p} <0.001$). A post-hoc Bonferroni correction indicated that the initial MVC ($\text {M}=100$, $\text {SD}=0.0$) was greater than the mid-game MVC ($\text {M}=80.2$, $\text {SD}=12.6$) and the post-game MVC ($\text {M}=76.2$, $\text {SD}=13.7$), and that the mid-game MVC was greater than the post-game MVC with $\text {p} <0.001$ for all comparisons. The same analysis was completed looking at males and females separately. For males there were differences between the pre-, mid- and post-game MVC $(\text {F}(2, 414)=196.5$, $\text {p} <0.001$) and a post-hoc Bonferroni correction indicated significant differences between all comparisons of pre-game MVC ($\text {M}=100$, $\text {SD}=0.0$), mid-game MVC ($\text {M}=80.7$, $\text {SD}=12.6$) and post-game MVC ($\text {M}=75.2$, $\text {SD}=14.1$) with $\text {p}<$0.001. Females showed differences between the three MVC values $(\text {F}(2, 291)=141.4$, $\text {p} <0.001$), with the post-hoc Bonferroni indicating that the initial MVC ($\text {M}=100$, $\text {SD}=0.0$) was greater than ($\text {p} <0.001$) the mid-game MVC ($\text {M}=79.4$, $\text {SD}=12.5$) and post-game MVC ($\text {M}=77.5$, $\text {SD}=12.9$) but also indicating no difference between the mid- and post-game MVCs.

### Properties of tracking error

**Fig. 9:**
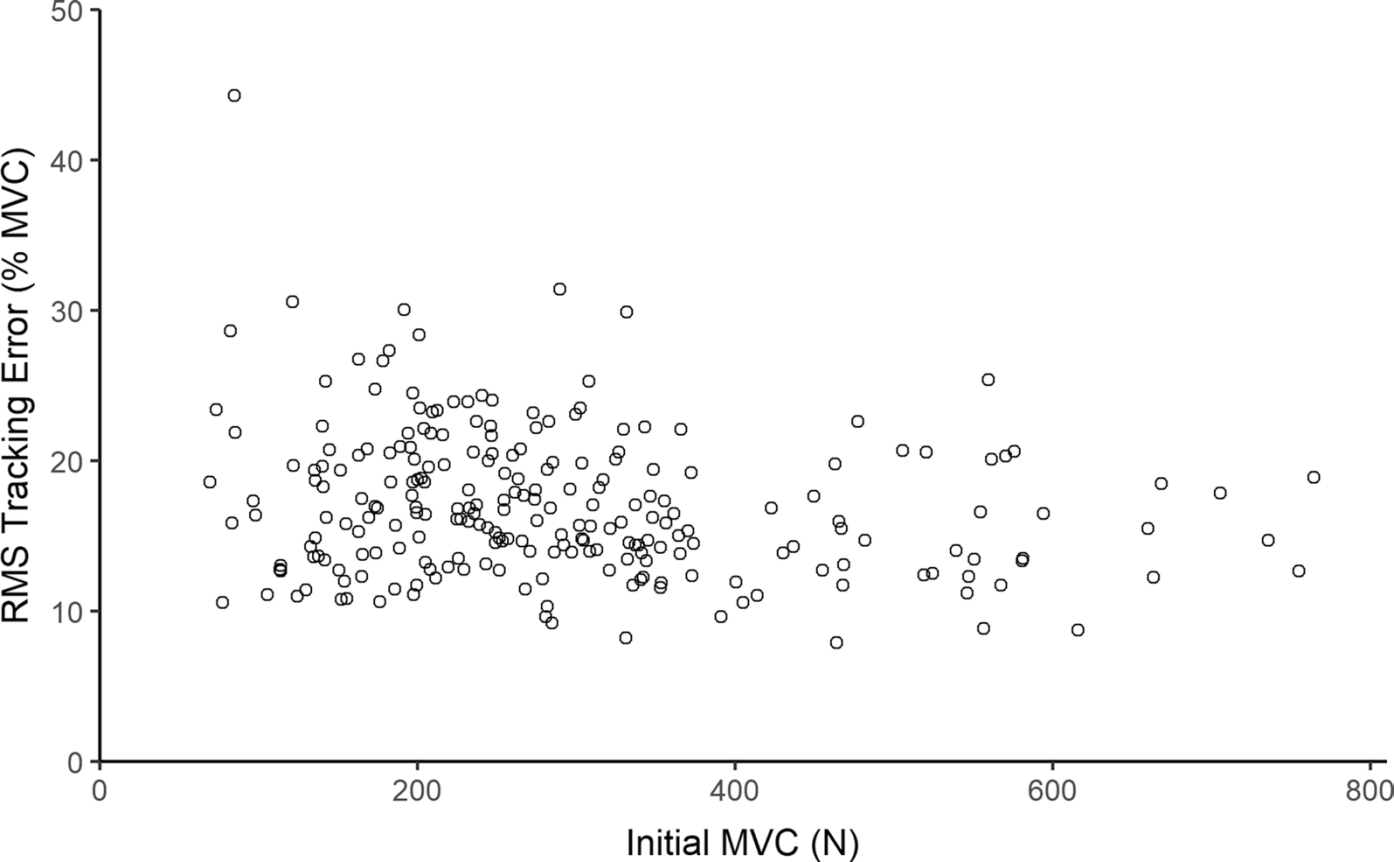
RMS tracking error over the entire game by MVC0 in a healthy population ($\text {N}=237$). The two variables are not strongly correlated, $\text {r}(235)=-0.22$, $\text {p} <0.001$

**Fig. 10:**
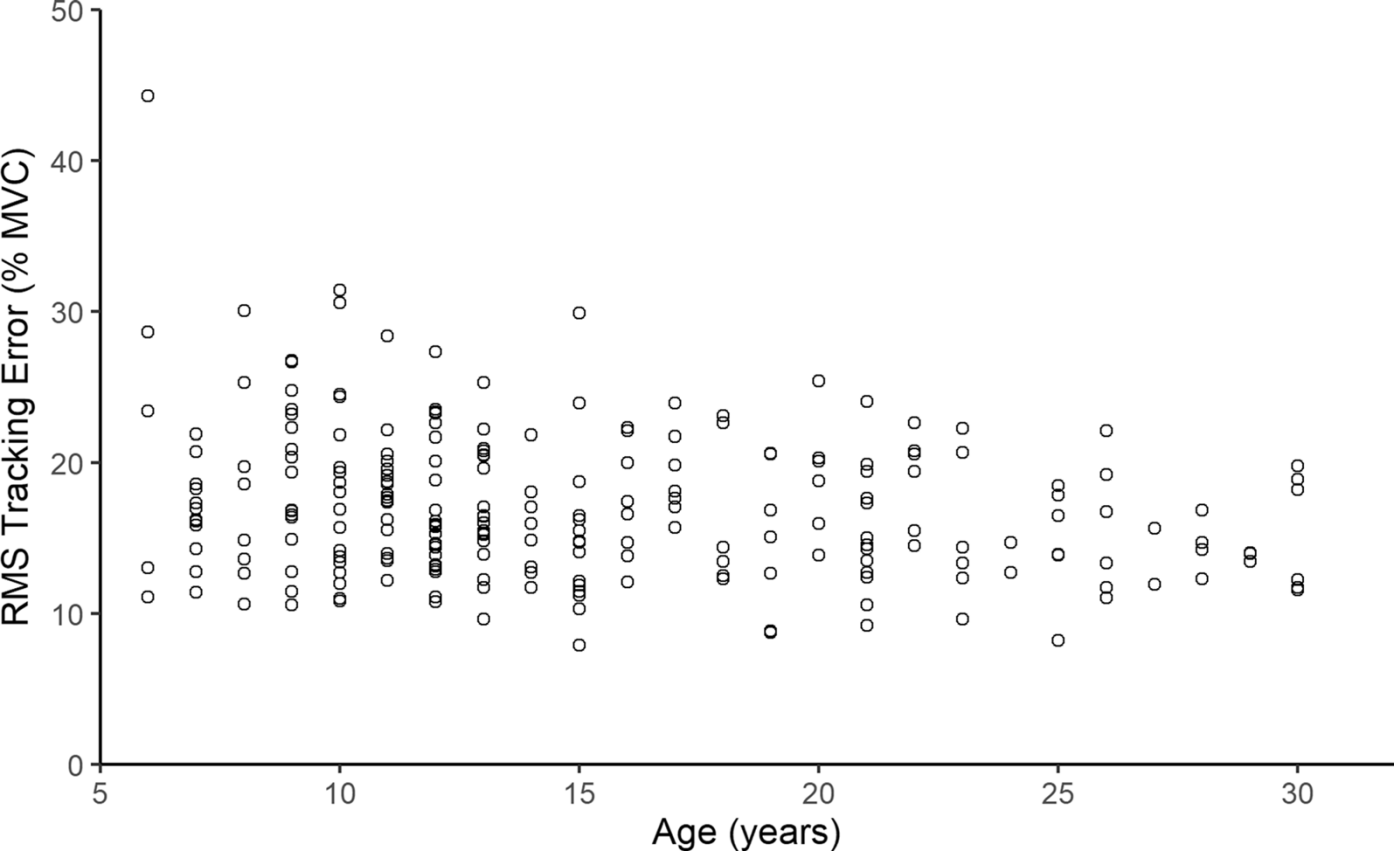
RMS tracking error over the entire game by age in a healthy population ($\text {N}=237$). The two variables are not strongly correlated, $\text {r}(235)=-0.22$, $\text {p} <0.001$

Because we believe that tracking error over time as the muscle fatigues may reveal useful diagnostic information about those with muscle disease, we needed to understand whether tracking error is affected by MVC or by age for the non-impaired population. Figure 9 shows how tracking error varied with MVC0 and Fig. 10 shows how tracking error varied with age. Both show weak correlations (Pearson’s $= 0.22$).

**Fig. 11:**
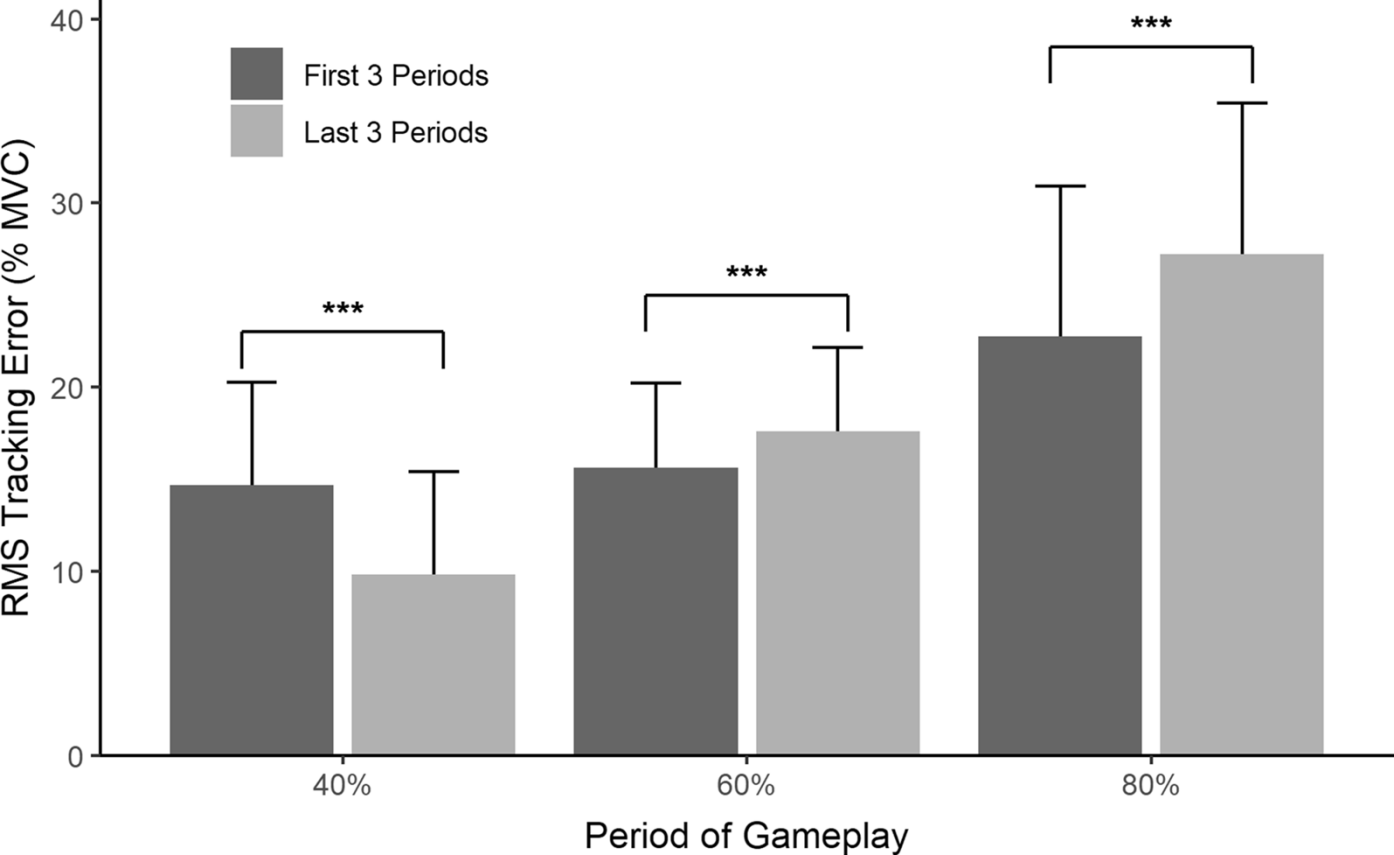
Compares RMS tracking error (mean + SD) for the 1st and 2nd 40, 60 and 80% of MVC0 target periods in a non-impaired population ($\text {N}=237$ in each group). For the 40% MVC0 target, tracking was better in the second half of the test while for the 60 and 80% MVC0 targets, tracking was worse in the second half

Participants’ tracking ability deteriorated at higher force levels as the game progressed. Figure 11 compares the RMS tracking error for 40, 60 and 80% of MVCO target periods during the first and second halves of the game. Tracking error decreased for the 40% periods $(\text {t}(236)=13.8$, $\text {p} <0.001$) but increased for the 60% $(\text {t}(236)=-5.2$, $\text {p} <0.001$) and 80% $(\text {t}(236)=-8.4$, $\text {p} <0.001$) periods.

Both genders experienced the increase in 80% MVC tracking error. Males increased from the 1st 80% MVC tracking period ($\text {M}=22.2$, $\text {SD}=8.8$) to the 2nd 80% MVC tracking period ($\text {M}=26.4$, $\text {SD}=11.3$), $(\text {t}(138)=-6.1$, $\text {p} <0.001$). Females increased from the 1st 80% MVC tracking period ($\text {M}=23.5$, $\text {SD}=7.1$) to the 2nd 80% MVC tracking period ($\text {M}=28.4$, $\text {SD}=10.8$), ($\text {t}(97)=-5.8$, $\text {p} <0.001$).

### Participants with muscle disease

**Fig. 12:**
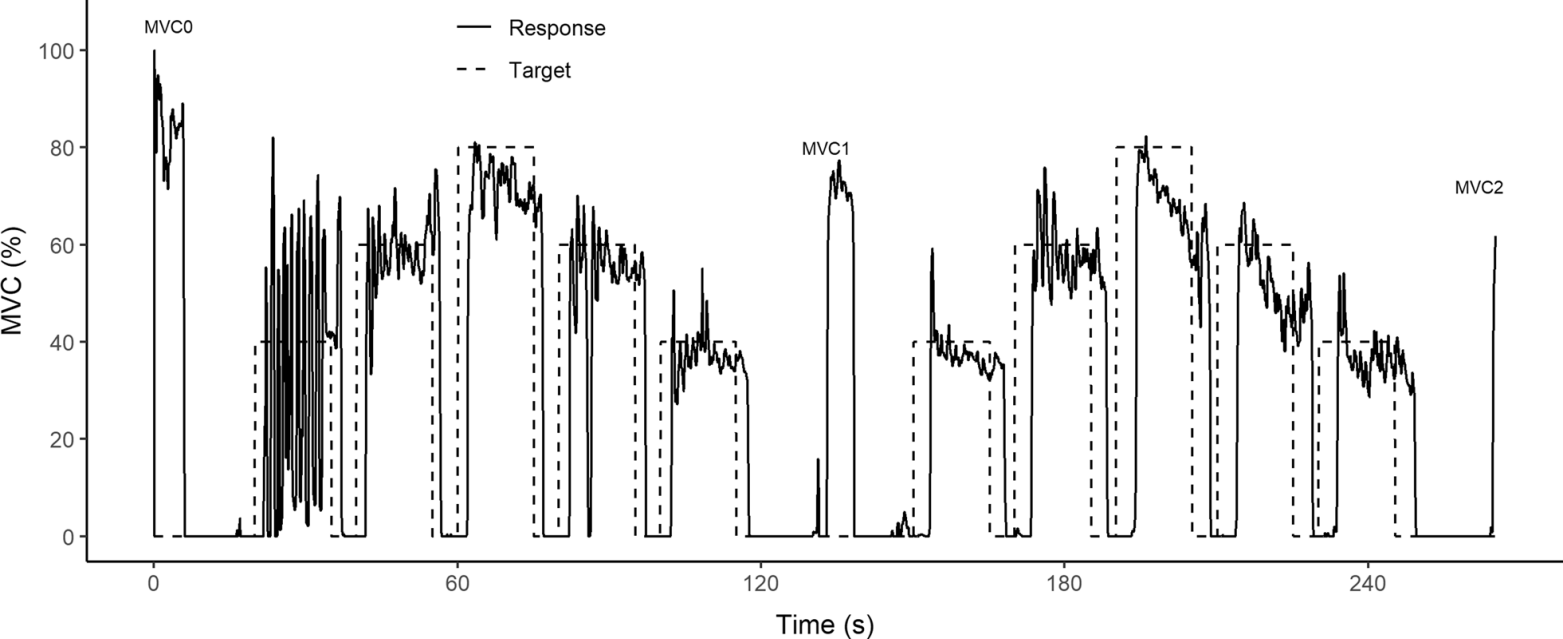
Example of run data from a 20 year old male participant with Duchenne muscular dystrophy

Thirteen participants with muscle disease (ages 6 to 20, 9 male, 4 female) were enrolled and were assessed using the new device during one of their normal visits to the University of Minnesota Muscular Dystrophy Clinic. Of the 13, eight were diagnosed with Duchenne muscular dystrophy, one with Becker muscular dystrophy, one with congenital muscular dystrophy and three with spinal muscular atrophy. Seven were ambulatory and six used a wheelchair. Of the 13, all but one completed the game. Figure 12 shows an example of dynamometer grip force data from a 20 year old male with Duchenne muscular dystrophy. As with many of the non-diseased participants, there were oscillations during the initial 40% tracking period as the participant was learning to control the rocket. For this participant, MVC dropped as the game progressed and fatigue is evident during tracking at the second-half 80% level.

## Discussion

The purpose of this study was to determine whether the hand grip device described in this paper produces grip strength measures that are consistent with normative data from the literature. A second purpose was to verify that participants are able to complete the fatiguing exercise protocol by playing a video game.

Because our hand grip system fatigues the user, we chose to compare the initial MVC values from our hand grip system to values reported in the literature rather than doing an experimental comparison to a traditional dynamometer such as the Jamar. If we had included a Jamar MVC in our measurements, the results would be contaminated by fatigue-induced order effects, particularly if participants played our fatiguing grip game first.

The results indicated that across males and females, 14 of 20 age groups had average MVC values that were the same as comparison data sets found in eight previous studies found in the literature. We chose to compare our data against all eight of the previous studies, including the widely cited studies by Mathiowetz [42, 43] because all eight used similar methods and all eight used a scientifically rigorous test protocol. The six age groups in our study that differed from the literature had differences that were small and in the same range as differences reported between previous studies. For example, when comparing data collected by Mathiowetz [42, 43] to six other studies using similar methods [49–54] we found that ten age groups had significantly different average MVC values. Because our device will primarily be used to track grip force changes over time in a single patient, any small differences in average MVC values found in our study compared to the MVC values found in the literature do not matter for the intended purpose of the device.

Unlike traditional hand grip tests, the new system takes the user through a more than four minute exercise protocol designed to induce fatigue. The reason for this is that we anticipate that fatigue profiles may reveal useful diagnostic information about children with neuromuscular disorders that goes beyond the information that can be gained from a single MVC reading. Our results for the pooled male and female data demonstrated that MVC decreased as the game progressed with maximum MVC at pre-game, a reduction at mid-game and the lowest at post-game, where MVC on average was 76% of the initial MVC. These results indicate that the exercise protocol did induce fatigue in a healthy population, a finding that was supported by talking with participants following their game session.

Tracking the higher force targets became more challenging during the second half of the game. For example, on average RMS tracking error increased by 19% during the second 80% MVC target period compared to the same target in the first half of the protocol. In contrast, tracking was better in the second half for the lower 40% MVC target (see Fig. 11). This was because most participants poorly tracked the first 40% target as they were learning to control the video game rocket by squeezing the dynamometer. For example, this is shown in the first tracking period in Fig. 4. This suggests that a brief get-to-know-the-game training period should be part of future protocols for diagnostic testing with the device.

At the higher force levels, the increased tracking error was mostly caused by muscle fatigue rather then by a loss of motor control. For the 80% target, 76 of the 237 participants were not able reach the first 80% target (average force of those 76 was 75% of MVC) while for the second 80% target, 135 participants were not able to reach the target (average force was 69%). At the 40% level the better tracking during the second period was a learning effect as participants become more skilled at manipulating their squeezing and even when fatigued were able to reach 40% of their MVC.

We found that the RMS tracking error was independent of participant age and initial MVC in a population that had no neuromuscular disease. This indicates that while the main diagnostic measure is force at the beginning and end of the test, RMS tracking error could be a useful supplemental metric for disease state diagnosis or for tracking the effectiveness of a treatment program for those with muscular dystrophy. The utility of tracking error as a metric that is independent of force won’t be known until data is collected from a large number of children with muscular dystrophy.

That all but one of the participants not only finished the game but also kept trying to earn points throughout the entire game, suggests that participants were motivated and engaged. (The one participant who did not finish the game put the controller down and walked away saying they did not want to continue playing the game). Because the game display kept a running total of points earned from observation it was clear that most participants were exerting their full strength during the calibration periods when MVC was recorded and during the tracking periods when RMS tracking error was recorded. Game points are normalized to initial MVC so that the total points scored is independent of absolute grip strength, which levels the playing field when participants play for points, providing additional motivation to engage.

While the main study used non-impaired participants by design, the intended patient population is children with neuromuscular disorders. While developing the prototype system, we conducted pilot tests using children with neuromuscular disorders and found that even children with weak grips were eager to play the game competitively against their non-affected siblings and parents. In addition, all but one of the 13 participants with muscle disease enrolled in the study reported here were able to complete the game indicating that the device is appropriate to assess muscle force in the intended patient population. The sample size of participants with muscle disease was too small to make any statements about changes in MVC or effects of fatigue. That information will be revealed when data collection from an ongoing clinical trial is complete.

There were several limitations to the study. First, it was difficult to determine in advance whether a participant should use the low-force or high-force dynamometer. Several were erroneously given the low-force dynamometer, which resulted in the large number (N=39) of data records that could not be used due to saturation of the force transducer. The age range of those who saturated the low-force system was 13 to 30 ($\text {M}=20.8$, $\text {SD}=4.7$), which suggests they should have been given the high-force system right away. To eliminate this problem, the next generation system will have a single hand grip and will have software that automatically adjust the gain, which will eliminate the force saturation problem. Second, due to ongoing revisions in the software, several ($\text {N}=27$) sessions had missing data and could not be used. Despite this, the data set used for analysis ($\text {N}=237$) was sufficiently large to validate the device. Third, while the dynamometer is lightweight, initial studies in patients with neuromuscular disease demonstrate that it is still too heavy for use by those with low arm and grip strength. We are currently revising the design so that the dynamometer is supported, which means the user does not have to support the weight of the device.

## Conclusion

Traditional measures of hand grip strength for monitoring muscle function in children with neuromuscular diseases offer no motivation for the child to exert maximum effort during the test and do not include a way to measure function as the muscle fatigues. The hand grip device described in this paper is intended to overcome both of these limitations. Our results show that the new device is valid when compared to normative data on hand grip forces reported in the literature. While the main study had non-impaired participants, from the initial results with the intended patient population we expect that the new device will be a reliable quantitative measuring tool for assessing muscle function in children with neuromuscular diseases including children with muscular dystrophy.

## Data Availability

The de-identified dataset analysed for the current study is available from the corresponding author on reasonable request.

## Abbreviations

DMD: Duchenne muscular dystrophy
MMT: Manual muscle test
QMT: Quantitative muscle testing
6MWT: Six-minute walk test
NSAA: North Star Ambulatory Assessment
PUL: Performance of the Upper Limb
MVC: Maximal voluntary contraction
MVC0: Beginning MVC
MVC1: Middle MVC
MVC2: Ending MVC
RMS: Root mean square
M: Mean
SD: Standard deviation
ANOVA: Analysis of variance
